# Mood Detection in Ambiguous Messages: The Interaction Between Text and Emoticons

**DOI:** 10.3389/fpsyg.2018.00423

**Published:** 2018-04-04

**Authors:** Nerea Aldunate, Mario Villena-González, Felipe Rojas-Thomas, Vladimir López, Conrado A. Bosman

**Affiliations:** ^1^Laboratorio de Psicología Experimental, Escuela de Psicología, Pontificia Universidad Católica de Chile, Santiago, Chile; ^2^Laboratorio de Afectividad y Comunicación, Escuela de Psicología, Universidad de Santiago de Chile, Santiago, Chile; ^3^Laboratorio de Neurociencia Cognitiva y Social, Facultad de Psicología, Universidad Diego Portales, Santiago, Chile; ^4^Cognitive and Systems Neuroscience Group, Swammerdam Institute, Center for Neuroscience, University of Amsterdam, Amsterdam, Netherlands; ^5^Facultad de Ciencias de la Salud, Universidad Autónoma de Chile, Santiago, Chile

**Keywords:** mood detection, disambiguation, text-based communication, emoticon, emotional valence

## Abstract

Face-to-face communication has several sources of contextual information that enables language comprehension. This information is used, for instance, to perceive mood of interlocutors, clarifying ambiguous messages. However, these contextual cues are absent in text-based communication. Emoticons have been proposed as cues used to stress the emotional intentions on this channel of communication. Most studies have suggested that their role is to contribute to a more accurate perception of emotions. Nevertheless, it is not clear if their influence on disambiguation is independent of their emotional valence and its interaction with text message valence. In the present study, we designed an emotional congruence paradigm, where participants read a set of messages composed by a positive or negative emotional situation sentence followed by a positive or negative emoticon. Participants were instructed to indicate if the sender was in a good or bad mood. With the aim of analyzing the disambiguation process and observing if the role of the emoticons in disambiguation is different according their valence, we measure the rate of responses of perceived mood and the reaction times (RTs) for each condition. Our results showed that the perceived mood in ambiguous messages tends to be more negative regardless of emotion valence. Nonetheless, we observed that this tendency was not the same for positive and negative emoticons. Specifically, negative mood perception was higher for incongruent positive emoticons. On the other hand, RTs for positive emoticons were faster than for the negative ones. Responses for incongruent messages were slower than for the congruent ones. However, the incongruent condition showed different RTs depending on the emoticons’ valence. In the incongruent condition, responses for negative emoticons was the slowest. Results are discussed taking into account previous observations about the potential role of emoticons in mood perception and cognitive processing. We concluded that the role of emoticons in disambiguation and mood perception is due to the interaction of emoticon valence with the entire message.

## Introduction

Social human interactions are based on emotional expressions. This information comes from several sources such as body gestures (Stekelenburg and de Gelder, 2004; Van Heijnsbergen et al., 2007), prosody (Heilman et al., 1984), or facial expressions (Haxby et al., 2002). The reason is that the affective information is crucial to understanding the others during the communicative situations (Pessoa, 2009). This issue has lead an important body of research to be focused on the emotional influence on human cognition, showing that human faces, for instance, are processed integrating their emotional valence (Krombholz et al., 2007; Lynn and Salisbury, 2008; Hinojosa et al., 2015), and the emotional context (Righart and de Gelder, 2008; Diéguez-Risco et al., 2015).

Nevertheless, nowadays digital technology has become one of the most used channels in human social communication. One of the technological options used in our interactions has been text-based conversations with instant messages. In this communication, information provided from face-to-face interactions is not available. This issue has been considered a factor that could hinder comprehension during text-based interactions (Kiesler et al., 1984). To compensate the lack of information in text-based communication, people use alternative pragmatic cues to enhance the emotional expressivity (Zhou et al., 2004; Hancock et al., 2007). One cue that has been extensively used to stress the emotional intentions on this channels has been the emoticon, which is considered a non-linguistic tool for the successful comprehension of messages (Walther and D’Addario, 2001; Kaye et al., 2016). Some authors have compared emoticons to non-verbal expressive gestures, suggesting that they replace them on text-based communication giving dynamicity to the conversations, and facilitating emotional emphasis (Feldman et al., 2017a,b). The function of emoticons in this dimension has yielded an incipient research to study their role in emotional comprehension during virtual communication (for a review see Aldunate and González-Ibáñez, 2017).

Most of the studies have focused their research on the active influence of emoticons in language comprehension. For example, it has been observed that interpreting the meaning in text-based messages is affected by the emoticons, which even facilitates the use of sarcasm in communication through the creation of ambiguities by manipulating its emotional valence (Derks et al., 2008). This issue is important because figurative language is characterized by ambiguity and it requires contextual information and strategies for its comprehension (Cornejo et al., 2007, 2009; Gibbs and Colston, 2012), which makes it more difficult to be understood in these channels. Other studies of sarcastic language comprehension have shown that emoticons have an important role for the design of machines to classify figurative language (Muresan et al., 2016). Filik et al. (2016) compared the effect of different emoticons (including non-face emojis) with the effect of punctuation marks on the comprehension of sarcastic messages, observing that emoticons were more expressive and effective for the disambiguation.

But language comprehension in human interactions requires knowledge of the interlocutors’ mood or emotional intentions in communicative acts because of their social character (Panksepp, 2004; Decety and Ickes, 2011). Lo (2008) studied the influence of emoticons on affective interpretation of messages. He asked participants to indicate on a scale the degree of happiness or sadness of messages without emoticons, and with congruent and incongruent emoticons. In the study it was observed that messages were interpreted as more positive when they were presented with happy emoticons, and that messages were considered more negative when they were accompanied by sad emoticons. Based on this, Lo (2008) concluded that emoticons contribute to a more accurate perception of the intentions of the text messages.

Although there are more studies about how emoticons help the comprehension of language, it has not been studied in depth the role that they play in the process of inferring the emotional state of the others. This point is necessary because it is known that cognitive and neural activity prioritize the emotional information influencing the processes (Haxby et al., 2002), but depending on the valence, because it has been observed that the integration of negative emotional information and positive emotional information is not the same at early stages of processing (Blau et al., 2007). The observed differences of the effect of the emotional valence in the processing implies that it is necessary to provide experimental evidence to observe if emoticons are independent of the valence of other sources in text-based communication, like linguistic information, or if they are prioritized in these channels of information. In this case, in ambiguous messages, we expect the emoticons to guide the inference of the mood of sender. On the other hand, if emoticons interact with other information present on messages, like the text’s emotional valence, during the disambiguation, we should expect differences in the inference of mood depending on the valence of both sources of valence information (text/emoticon).

In the present study, we designed an experiment to investigate the influence of the valence of the text in the influence of the emoticons to disambiguate messages on the inference of sender’s mood. Using an emotional decision task in an affective congruence paradigm, we presented text-based messages with positive and negative valence, followed by typographic emoticons which could have positive and negative valence. Our aim was to analyze the influence of emoticon valence on the capacity to infer the interlocutor’s mood and on the reaction time (RT) in order to observe if the emoticon is the prioritized source of information, or if it depends on the affective contextual information in which it appears.

## Materials and Methods

### Participants

Seventy-four undergraduate students participated on the experiment (Mean age = 21.3; ± 1.93 SD; 52 women). All of them were native speakers, and none had psychiatric disorders or neurological diseases. All the participants signed an informed consent form in accordance with the Declaration of Helsinki, which was authorized by the Institutional Ethical Committee.

### Materials

A list of 60 sentences (Supplementary Table S1) was created describing an emotional situation uttered by a person (**Table 1**). Thirty sentences were about positive situations and 30 were about negative situations. The complete set of sentences was previously validated to control the equivalence in emotional intensity between both lists of situational sentences (positive vs. negative). Stimuli validation was carried out through the administration of a questionnaire with all the situational sentences. Sixty-eight participants (49 women, Mean age: 22.9 years ± 0.7 SD) were instructed to indicate the valence and the emotional intensity with a Likert scale of response where one meant very negative, and five meant very positive. Both lists of situational sentences were statistically similar in emotional intensity [*t*(58) = 1.062; *p* = 0.290], with a comparable mean of intensity of valence for positive (Mean = 4.6; ± 0.3 SD) and negative (Mean = 4.5; ± 0.3 SD).

**Table 1:** Examples of positive and negative situational sentences.

Positive situational sentences	Negative situational sentences
Me regalaron un auto nuevo	Me chocaron el auto
(*They gave me a new car*)	(*They crashed my car*)
Me felicitaron en el trabajo	Me echaron del trabajo
(*They congratulated me at work*)	(*I was fired*)
Entregué mi tesis	Se borró mi tesis
(*I handed in my thesis*)	(*My thesis was deleted*)
Tengo una pareja hermosa	Mi novio me va a dejar
(*I have a wonderful partner*)	(*My boyfriend is going to break up with me*)

**FIGURE 1:**
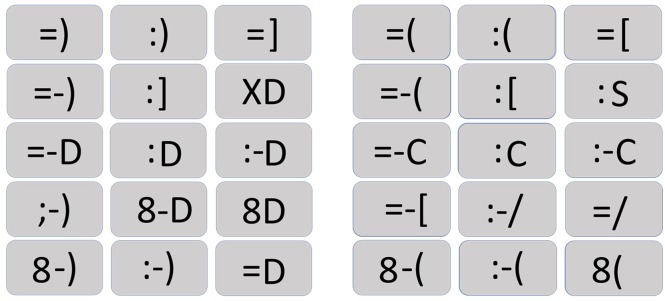
Typographic emoticons set. The 15 positive emoticons used on the experiment are on the left side of the figure, and the 15 negative emoticons are on the right side.

For the experiment were used a set of 30 typographic emoticons (**Figure 1**) with Font Calibri (15 positive emoticons and 15 negative emoticons). Both lists were controlled by emotional intensity, through the administration of a questionnaire were participants had to indicate the valence and emotional intensity for each one. 75 participants (Mean Age = 20.46; *SD* = 0.4; 47 women) complete the questionnaire in a Likert scale, where 1 meant very negative, and 5 meant very positive. Both sets of emoticons (positive vs. negative) presented the same emotional intensity [*t*(28) = 1.193; *p* = 0.24], with a comparable mean of intensity of valence for positive (Mean = 4.4; ± 0.3 SD) and negative (Mean = 4.3; ± 0.4 SD).

### Procedure

In laboratory, all the participants were handed an informed consent form, which all signed after being briefed on the conditions and instructions of the experiment. They had to sit in front of a screen and they were instructed to respond in each trial with a button if they considered if the person that wrote the message was with a good or bad mood at the time he/she sent it. In the first half of the task, subjects pressed a right bottom when they consider the message was written in a good mood. Conversely, subjects were prompted to response with a left bottom when they consider the message was written in a bad mood. To control lateralized motor response effect, we counterbalanced response buttons after the first half of trials. Subjects were instructed to respond as fast as possible once they perceive the emotional content of the message.

**FIGURE 2:**
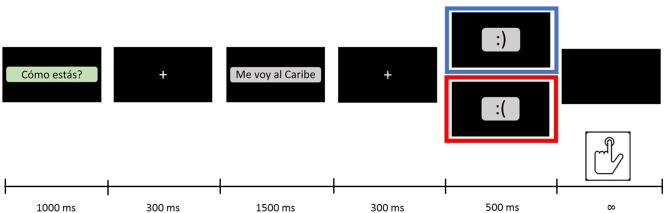
Experimental trial. Sequence of events on each trial. Time of presentation is represented in milliseconds (ms). Blue corresponds to congruent emoticon, and red corresponds to incongruent emoticon.

Psychopy software (Peirce, 2007) was used to build and present the experiment. Trials simulated a brief text-based conversation where a person responded to the question “How are you?” with a situational sentence finishing with a typographic emoticon (**Figure 2**). Each trial began with the text-based message asking “How are you?” presented 1,000ms on a black background. After that, it was presented the situational sentence during 1,500ms on the screen. Finally, the typographic emoticon was presented on screen during 400ms. Participants had no time restrictions to respond for each trial, but they were instructed to do it as fast it was possible. Once they press some of the response buttons, next trial initiated. The reason for the order of each trial (first text; second emoticon) was to maintain the structure of the most frequent text-based communication (Novak et al., 2015).

To avoid buildup, all trials were randomized controlling the sequence of trials presentation belonging to the same conditions. Randomization considered the following criteria: (a) trials with the same condition of affective congruence (valence x congruence: pp / nn / np / pn) could not be presented more than twice in a row; (b) trials with the same congruence condition (congruent / incongruent) could not be presented more than three times in a row; (c) trials with the same valence in situational sentence could not be presented more than three times in a row; (d) trials with the same emoticons’ valence could not be presented more than three times in a row; and finally, (e) trials with the same emoticon could not be presented twice in a row.

### Statistics

All statistics were done using STATISTICA 7.0 software (StatSoft, Inc). We performed a dependent samples *t*-test to compare the amount of trials in which participants judged as good or bad mood in the different conditions. Repeated-measures ANOVA was performed to compare RTs. We used congruency (levels: congruent, incongruent), emoticon valence (levels: positive, negative) and response (levels: good mood, bad mood) as factors.

## Results

**FIGURE 3:**
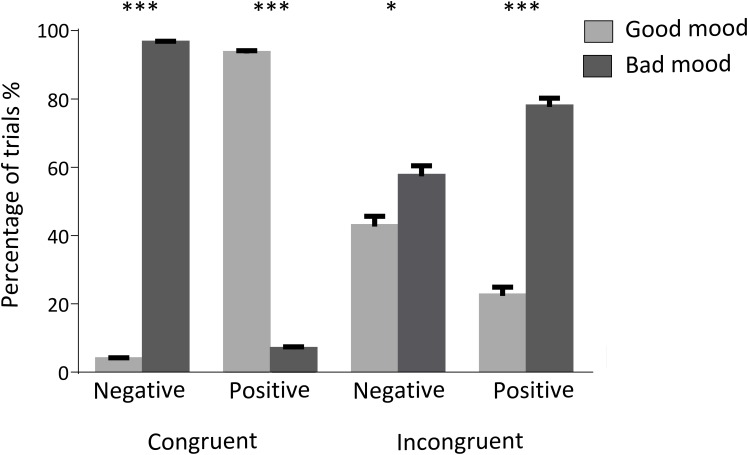
Percentage of trials judged as good or bad mood. Bars indicates the mean and SEM of the percentage of trials judged as good or bad mood for each condition. ‘Negative’ and ‘Positive’ indicates the emoticon’s valence. ^∗^, ^∗∗∗^: statistically significant differences with *p* values < 0.05, or < 0.001, respectively.

We measured mood selection by calculating the number of trials which participants judged as good or bad mood in the different conditions (**Table 2**). Differences can be observed for each condition. Specifically, participants judged as bad mood in the negative congruent condition (*t*(78.89), *p* < 0.01) and good mood in the positive congruent (*t*(55.17), *p* < 0.01). On the other hand, participants judged as bad mood in the incongruent conditions regardless of the emoticon emotional valence. However, the difference observed in the negative incongruent condition (good mood = 12.789, bad mood = 17.211; *t*(2.423), *p* = 0.018) is notoriously smaller than the positive incongruent condition (good mood = 6.704, bad mood = 23.296; *t*(10.79), *p* < 0.01) (**Table 2** and **Figure 3**).

**Table 2:** Descriptive behavioral results. The table shows the selection response rate (%), according to whether participants perceived a good or bad mood of senders, and Reaction times (RT), for each condition (NN: Negative Congruent; PP: Positive Congruent; NP: Positive Incongruent; PN: Negative Incongruent). For all results, Standard Deviation (SD) and Standard Error (SEM) are indicated.

		GOOD MOOD		BAD MOOD	
		Frequency	RT	Frequency	RT
NN	MEAN	1,099 (3,662%)	1,138	28,901 (96,338%)	1,028
	SD	1,485	0,621	1,485	0,404
	SEM	0,176	0,074	0,176	0,048
PP	MEAN	28,000 (93,333%)	1,065	2,000 (6,667%)	1,502
	SD	1,986	0,429	1,986	0,902
	SEM	0,236	0,051	0,236	0,107
NP	MEAN	6,704 (22,347%)	1,636	23,296 (77,653%)	1,467
	SD	6,477	0,886	6,477	0,651
	SEM	0,769	0,105	0,769	0,077
PN	MEAN	12,789 (42,629%)	1,872	17,211 (57,371%)	1,878
	SD	7,688	1,280	7,688	1,102
	SEM	0,912	0,152	0,912	0,131

We measured task performance by calculating the RTs for each condition. We compared them in order to assess whether there are interactions between the emotional congruence between a text message and the subsequent emoticon stimulus, the valence of the emoticon and mood detection, and how these dimensions influence behavioral response.

**FIGURE 4:**
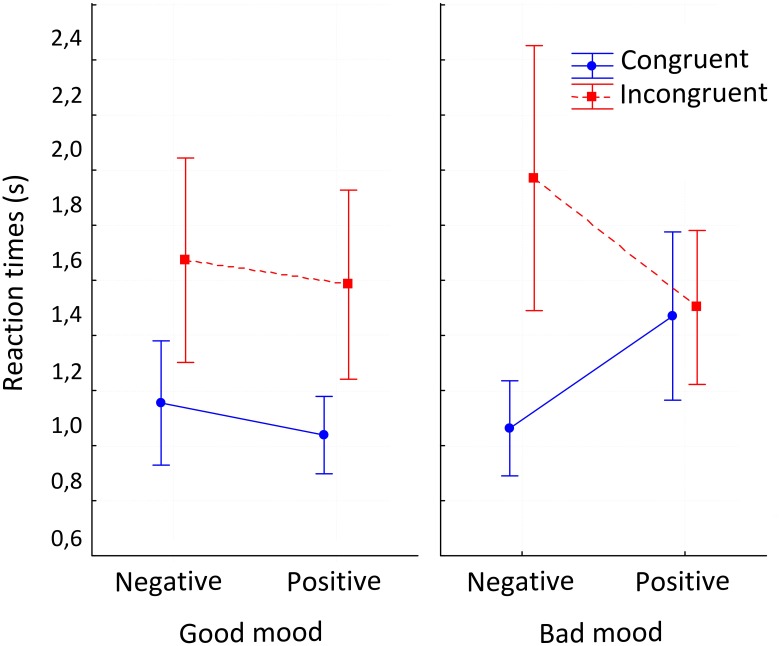
Interaction between the three factors. Interaction effect in repeated measure ANOVA between congruency, emoticon’s valence and mood detection factors. Vertical bars denote 0.95 confidence intervals.

We found a main effect for mood detection, where “bad mood” showed slower RTs than “good mood” (*F*(1,31) = 7.435; *p* = 0.010). We also found an effect for congruency factor, where incongruent condition showed slower RTs than congruent condition [*F*(1,31) = 19.27; *p* < 0.01]. Valence factor showed no differences [*F*(1,31) = 1.217; *p* = 0.278] (**Figure 4**).

Furthermore, an interaction effect between two factors (congruence/valence) was observed [*F*(1,31) = 10.43; *p* < 0.01]. Specifically, during incongruent conditions, negative emoticons had slower RTs than positive emoticons. We also found an interaction between the three factors (congruency, valence and mood detection) [*F*(1,31) = 16.56; *p* < 0.01]. Specifically, when participants selected good mood response, incongruent condition showed slower RTs contrasted with congruent condition, regardless of the valence of emoticon. On the other hand, when they selected bad mood response, the positive emoticon showed the same RT for both congruent and incongruent. However, when emoticon valence is negative, incongruent condition showed slower RTs contrasted with congruent condition (**Figure 4**).

## Discussion

The current study analyzes the role of emoticons to disambiguate messages to detect the state of mood of the sender during a text-based communication. We used an affective congruence task where participants had to select if the sender was on a good or bad mood, reading text-messages describing emotional situations with a critical emoticon at the end. We manipulated the emotional valence of the emoticons, generating congruent and incongruent affective messages. Our focus was in the incongruous conditions to observe which information is used by the participants to detect emotions. Our results indicated that the emoticons did not always guide the disambiguation processes on text-based interactions. Specifically, we observed that the ambiguous messages (incongruent condition) with positive emoticons (negative text) and with negative emoticons (positive text), are selected more frequently as messages sent by persons with a negative state of mood. In addition, our results show that there are interactions between the valence of the emoticon and the valence of the preceding text. In spite of the tendency to select the negative mood, this tendency differs if we analyze the different types of ambiguities. Specifically, in conditions where emoticons had a positive valence in incongruent messages, the rate of selection as good or bad mood was more similar than when the incongruent condition ended with a negative emoticon. In this case, the tendency to select bad mood was higher than the incongruent condition which presented positive emoticons, with a greater difference of response rates selecting good or bad mood. Additionally, this tendency is inverted, observing that incongruent messages with negative emoticons tend to be related to good emotional states than when the incongruent messages ended with a positive emoticon.

On the other hand, a congruence effect was observed on the RTs when the participants selected good mood response, with slower RT for incongruent messages; but this effect disappeared when the participants selected bad mood response. Furthermore, the congruence effect was observed only when emoticons were negative with slower RT in incongruent condition. Additionally, negative emoticons had slower RTs than positive emoticons.

In the following we will further discuss some of these points.

### Ambiguous Messages Tend to Be Perceived as Bad State of Mood of the Sender, but This Tendency Depends on the Valence of the Emoticon

Results of this experiment show that ambiguous messages are more frequently associated with a bad mood of the sender, but also that this difference is more pronounced when emoticons had a positive valence. This evidence is contradictory with results obtained in other studies, which suggests that emoticons valence guides the mood perception being prioritized to detect emotions in text-based communication (Derks et al., 2007). Lo (2008), with a task similar to the one set out in this study, presented different conclusions from those obtained from our results. Lo (2008) observed that the valence of the emoticon guided the perception of mood; while we observed that in the incongruent conditions, the influence of emoticons on the disambiguation of incongruous messages for the perception of mood is not the same when the emoticons are positive or negative.

Some distinctions can be crucial to explain the differences between both studies. Stimuli used by Lo (2008) were presented during 1 min, while in our study emoticons were presented during 500 ms. This implies that when participants have less time to respond to select the mood of the message, they do it with less deliberated inference processing. Longer times to select responses could facilitate a reflexive analysis for judging the mood; while we obtained results of earliest stages of mood detection process. Additionally, Lo (2008) instructed the participants to indicate the degree of sadness or happiness perceived on each messages. Our responses were dichotomic alternatives, where participants had to indicate only if they perceived a good or bad mood. This difference is related with the previous point (i.e., the deliberation), because, to indicate the degree of a specific emotion intensity, implies analyzing the stimuli which is a more complex process. In our experiment, we did not take the level of perceived emotional intensity, but, with the previous validation of stimuli, we controlled that the observed valence effects in ambiguous messages, were not due to differences on emotional intensity.

The observed interaction between the emoticons and the ambiguity suggests that considering text and emoticon have dissimilar and isolated functions – text communicating ideas, and emoticons communicating emotions (Knapp and Hall, 2002) – is not an appropriate approach. Our results imply that it is more plausible to consider that their role (text and emoticon) has to be considered from the relationship between both of them to detect sender’s mood from ambiguous messages.

One aspect that has been proposed about emoticons is that these cues provide enjoyment to the interaction (Huang et al., 2008). Recording electrodermal activity to measure arousal, and electromyography to measure facial micro-expressions, Thompson et al. (2016) observed that sarcastic expressions (which by definition are ambiguous) presented more positive responses when they were accompanied by emoticons, increasing arousal levels and enhanced smiling. Considering the observations of Thompson et al. (2016) and the fact that we observed that people tend to detect negative emotions from ambiguous messages, make us to assume that although we enjoy conversation when emoticons are included, we are detecting the negative emotions in the messages.

Our results are not the first to show the negative emotions detection tendency in ambiguous messages. Similarly, Walther and D’Addario (2001) found that when messages contained any negative information (in text or emoticon) there was a higher tendency to perceived negative emotions. These findings, including ours, indicate that considering an isolated influence of emoticons is a limited explanation when messages are ambiguous. Rather, we propose that it has been considered that the emoticon’s functionality has to be related with a role on the indication of illocutionary force on text-based CMC (Dresner and Herring, 2010, 2014). Thus, from a Speech Act Theory (Austin, 1975; Searle, 1985), emoticon would have a pragmatic meaning, with a function which goes beyond its emotional information, similar to what happens with punctuation marks or even gestures (Feldman et al., 2017b).

### Future Directions

To our knowledge, this is the first study where the detection of emotions in ambiguous text-based communication is experimentally measured from the RTs, and not just from a selection response. Ambiguity has been a difficulty for CMC, not just for human interactions, but also to create computational algorithms to sentiment analysis. Example of this is irony or metaphorical language, which are part of the figurative language in opposition to literality (Sarbaugh-Thompson and Feldman, 1998). With our results, we can see that disambiguation not only depends on the emoticon, but also on the interaction with its contextual information. Nevertheless, further experimental research is required to understand the active role on disambiguation of emoticons in their context.

This study did not control the perceived irony of the expressions after every message, which is undoubtedly one of the ways of making sense of ambiguous sentences (Cornejo et al., 2007). Therefore, further designs should also control for the perceived irony. In addition, previous studies have observed that virtual interactions present individual differences according to personality traits like extraversion or openness to experience, which could be influencing on the user’s perception in virtual communication (Kaye et al., 2016, 2017). We did not measure personal traits which could be influencing the detection of mood states from text-messages related with social interactions (receiver’s mood; empathy levels, communicative skills or social skills). It would be useful to observe if some of these traits could predict personal differences on the tendency to infer emotional states of others on communicational interactions. Additionally, experimental manipulations of factors, which is known to influence comprehension, like for example, age, gender, cultural differences, information knowledge speaker or common ground, among others (Kronmüller and Barr, 2007; Van Berkum et al., 2008; Galati and Brennan, 2010), would allow to understand emotional detection of sender and similarities and differences between text-based communication and face-to-face communication.

Regarding this study, it is necessary to bear in mind that this experiment used typographic emoticons to assess the question of their role on emotional disambiguation in text-based communication. These cues are schematic symbols and not complex images like emojis, which have more details on the emotional expressions. Differences in their influence on the emotional expressivity has been observed on previous studies (Nobuyuki et al., 2014), so it is important to consider this difference on new researches.

Finally, this experiment uses trials initiated by text and not observed if the order of the text and emoticons had another effect into comprehension, but it is known that text + emoticons is the more usual structure of messages (Novak et al., 2015). Nevertheless, to study if there is a difference in results due to the order of the composition of each trial could help to control emotional priming effects influenced by the way in which stimuli are presented (text vs. emoticon) (Hermans et al., 1994; Stenberg et al., 1998; Spruyt et al., 2002).

## Conclusion

Emoticons have been considered the expressive cue used in text-based messages to enhance the intended emotion in CMC. Nevertheless, future theories and studies have to consider the contextual influence to understand the emoticons processing in CMC. It is known that human communication requires the integration of all available contextual information (Tanenhaus et al., 1995; Xu et al., 2005). For example, gestures and body movements are integrated to the speech processing (Willems et al., 2006; Özyürek et al., 2007; Cornejo et al., 2009). Our results indicate that this also occurs in the virtual text-based communication, integrating text and emoticon information for detecting the mood of the sender on affective communication. Furthermore, we conclude that this integration depends on the valence of the information on the disambiguation of messages, which seems to be more difficult in the conditions were the incongruity contains a positive text and a negative emoticon. All this leads us to emphasize the need to study the role of emoticons taking into account their relation to the context in which they are presented, and not as isolated information. All this leads us to emphasize the need to study the role of emoticons taking into account their relation to the context in which they are presented, and not as isolated emotional information, considering that communication is a complex phenomenon. Specifically, we observed from our study that it is important to consider emoticons from their pragmatic function in CMC.

## Author Contributions

NA, MV-G, FR-T, VL, and CB made substantial contributions for the conception and design of the work, drafting and revising this manuscript critically, and approving the final version to be published. All of them agreed to be accountable for all aspects of the work ensuring that any question related to the work are appropriately investigated and resolved.

## Conflict of Interest Statement

The authors declare that the research was conducted in the absence of any commercial or financial relationships that could be construed as a potential conflict of interest.
