# Supplementary material for: Mood Detection in Ambiguous Messages: The Interaction Between Text and Emoticons
Source: Front Psychol. 2018 Apr 4;9:423. doi: 10.3389/fpsyg.2018.00423 (PMC5893907; doi:10.3389/fpsyg.2018.00423)
Supplement: Supplementary file 1 [file Table_1.PDF]

LIST OF POSITIVE AND NEGATIVE SITUATIONAL SENTENCES

Positive Situational Sentences

Conocí a alguien muy especial

Dijeron que mi idea es la mejor

Dormí muy rico anoche

Entregué mi tesis

Esta mañana, nació mi hijo

La fiesta estuvo buenísima

Me ascendieron en el trabajo

Me dijo que me quería

Me encantó el regalo que me hiciste

Me felicitaron en el trabajo

Me fue súper bien en el examen

Me gané unas entradas para ver a mi grupo favorito

Me regalaron un auto nuevo

Me saqué un 7,0 en la prueba de álgebra

Me subieron el sueldo

Me voy al Caribe

Mi familia está más unida que nunca

Mi guagua es hermosa

Mi mamá se ganó la lotería

Mi salud es excelente

Nunca había tenido tanta suerte

Pasé un excelente fin de semana junto a mi familia

Quedé primero en la carrera

Salí de vacaciones

Son mis mejores amigos

Tengo los mejores amigos del mundo

Tengo una pareja hermosa

Tomé la mejor decisión de mi vida

Voy a comer mi comida favorita

Ya tengo los pasajes para mi viaje

Negative Situational Sentences

Cada vez tengo más estrés

En mi casa están todos en contra mío

Esa familia no tiene con qué comer

Este mes no me pagaron

Estoy pasando un momento muy difícil

Hoy es mi cumplaños y nadie se acordó

Me chocaron el auto

Me dijeron que tengo una enfermedad grave

Me duele mucho la cabeza

Me echaron del trabajo

Me fracturé un tobillo

Me pusieron una multa

Me robaron la bicicleta

Me saqué un 1,0 en la prueba de historia

Me traicionaron

Mi jefe me humilló en público

Mi novio me va a dejar

Mi papá se cayó por las escaleras

Mi pareja me dejó

No creo que logre la nota necesaria para pasar

No encuentro mi billetera

No tengo dinero para comer hoy

No tengo ningún amigo de verdad

Perdí el avión

Reprobé casi todos los exámenes finales

Se borró mi tesis

Se me rompió el computador

Se murió un familiar cercano

Se perdió mi perro

Tengo mucho trabajo atrasado
